# Exploring the Potential Molecular Mechanism of *Scutellaria baicalensis* Georgi in the Treatment of Gastric Cancer Based on Network Pharmacological Analysis and Molecular Docking Technology

**DOI:** 10.3389/fphar.2021.697704

**Published:** 2021-08-06

**Authors:** Yi Tu, Quanli Wu, Jiarui He, Jiasheng Xu, Shasha Yu, Qingfei Wang, Yunqi Cheng, Qijun Yang, Shan Xu, Yi Cao

**Affiliations:** ^1^ Department of Pathology, The First Affiliated Hospital of Nanchang University, Nanchang, China; ^2^ School of Public Health, Nanchang University, Nanchang, China; ^3^ Department of Basic Disciplines, Jiangxi Health Vocational College, Nanchang, China; ^4^ Leping Hospital of Traditional Chinese Medicine, Jiangxi Province, Jingdezhen, China; ^5^ Queen Mary College of Nanchang University, Nanchang, China; ^6^ Department of Gastrointestinal Surgery, The First Affiliated Hospital of Nanchang University, Nanchang, China

**Keywords:** *scutellaria baicalensis* georgi, gastric cancer, network pharmacological analysis, molecular docking technology, ESR1, FOS

## Abstract

**Objective:** To explore the molecular mechanism of *Scutellaria baicalensis* Georgi in treating gastric cancer by network pharmacological analysis and molecular docking.

**Methods:** Taking *Scutellaria baicalensis* Georgi as the object, the active components and corresponding potential drug targets in *Scutellaria baicalensis* Georgi were obtained from the database of TCM Pharmacological System Analysis Platform (TCMSP). GeneCards/OMIM/DrugBank and other databases were used to collect gastric cancer-related genes, and the obtained genes were intersected with drug targets to obtain the target genes of *Scutellaria baicalensis* Georgi on gastric cancer. Furthermore, the interaction network of *Scutellaria baicalensis* Georgi-active ingredients-target-gastric cancer-related genes was constructed. Protein–protein interaction analysis and gene ontology (GO) and Kyoto Encyclopedia of Genes and Genomes (KEGG) enrichment analysis were performed on target genes. The PubChem website was used to screen the compounds corresponding to the target genes, and the target protein and 3D structure pdb format files were obtained from the PDB database. Finally, the molecular docking calculation was performed by the AutoDock Vina program. The *in vivo* cell experiments on the effect of *Scutellaria baicalensis* on proliferation and migration of gastric cancer cells were used to determine the therapeutic effect of *Scutellaria baicalensis* on gastric cancer, and the two genes ESR1 and FOS are the key targets of *Scutellaria baicalensis* on gastric cancer.

**Results:** A total of 10 gastric cancer-related target genes were screened out, and *Scutellaria baicalensis* Georgi contained 10 active compounds targeting 10 gene sites. There are 30 effective compounds in *Scutellaria baicalensis* Georgi targeted to treat gastric cancer, and there are 91 corresponding targeting gene sites, involving a total of 10 pathways. The results of molecular docking show that ESR1, FOS, and *Scutellaria baicalensis* Georgi have good binding free energy and docking fraction. The docking fraction of FOS is −4.200 and the binding free energy is −27.893 kcal/mol. The docking fraction of ESR1 is −5.833 and the binding free energy is −30.001 kcal/mol. The effect of *Scutellaria baicalensis* Georgi on gastric cancer was verified by *in vitro* cell experiments and Western blotting.

**Conclusion:***Scutellaria baicalensis* Georgi can target and regulate multiple signal pathways by acting on ESR1 and FOS gene loci, thus having a potential therapeutic effect on gastric cancer.

## Introduction

In recent years, due to changes in diet and living habits, the incidence of gastric cancer has been maintained at a high level and has gradually become a cancer type that seriously threatens human health and life. Gastric cancer is a malignant tumor of the digestive tract with high morbidity and mortality. According to the recently released cancer research report, it is estimated that there will be nearly 680,000 new cases of gastric cancers and nearly 500,000 deaths in China, ranking second in both morbidity and mortality (Tan and Li, 2020). At present, surgical resection is still the most effective way to treat patients with early gastric cancer. However, most patients were diagnosed late, and the postoperative recurrence and mortality were still high despite the operation. The 5-year survival rate of patients with advanced gastric cancer is only 30.4% (Wang et al., 2020). The occurrence and development of gastric cancer is a complex process involving multiple pathways and multiple genes. It is particularly important to clarify the root causes of gastric cancer and find innovative treatment strategies for the treatment of gastric cancer.

Natural medicines have the advantages of small side effects and diverse biological potency. Many active ingredients of traditional Chinese medicines can achieve the antitumor effect by inducing autophagy and apoptosis of gastric cancer cells, thus providing a new breakthrough point for the treatment of gastric cancer (Lee et al., 2019; Huang et al., 2020; Zhang et al., 2020).

*Scutellaria baicalensis* Georgi is the root of *Scutellaria baicalensis* Georgi of Labiatae, and baicalein is a natural compound extracted from *Scutellaria baicalensis* Georgi of Labiatae, which is often used to treat constipation, abdominal distention, cough, and other symptoms of gastrointestinal patients in the clinic. Many studies have shown that (Wang et al., 2018a; Cheng et al., 2018; EghbaliFeriz et al., 2018; Zhao et al., 2019a; Limanaqi et al., 2020) scutellarin plays a variety of physiological and pharmacological effects in anti-inflammatory, antiviral, and antitumor. In addition, studies have shown that *Scutellaria baicalensis* Georgi has a good effect in inhibiting gastric cancer, and it has a good adjuvant treatment effect on patients with advanced gastric cancer. In recent years, it has been found that *Scutellaria baicalensis* Georgi can inhibit the proliferation of tumor cells, while baicalin has different inhibitory effects on malignant tumor cells such as liver cancer, colon cancer, breast cancer, and lung cancer and induces apoptosis of tumor cells in a dose-dependent manner.

Various studies have shown that baicalin can effectively inhibit the invasion, migration, and adhesion of various tumors *in vitro* by inhibiting the expression level and activity of MMP2 and MMP9. Baicalin inhibited the migration and invasion of HeLa and SiHa cells in a dose-dependent manner (Wang et al., 2018b). It can inhibit protein kinase c/signal transducer and activator of transcription 3 signal transduction in cervical cancer cells and downregulate protein expression levels of STAT3 signal pathway target genes such as MMP2, MMP9, and survivin in a concentration-dependent manner. In addition, baicalin can also inhibit the migration and invasion of cervical cancer HeLa cells by downregulating the expression levels of MMP2 and MMP9 through the p38-MAPK pathway and has a concentration-dependent effect to a certain extent (Zhang et al., 2016a; Wang et al., 2018b). Researchers have applied a series of transcriptome analysis methods to determine the candidate anticancer molecular mechanism of *Scutellaria baicalensis* Georgi. They found that multichannel interference and cell cycle recognition are the potential ways for *Scutellaria baicalensis* Georgi to inhibit the proliferation of gastric cancer cells, which may inhibit the migration of gastric cancer cells and induce the apoptosis of gastric cancer cells through NF-kB and other signal pathways (Wang et al., 2016; Wang, 2017; Lin et al., 2019). The gene expression pattern triggered by *Scutellaria baicalensis* Georgi affects the same pathway as chemotherapy, but it affects different genes. While these genes regulate cell division and death cycle, *Scutellaria baicalensis* Georgi may change the way of regulating the cell cycle and push cancer cells toward the path of cell death, thus killing cancer cells (Zhang et al., 2016b; Lin and Huang, 2019).

*Scutellaria baicalensis* Georgi can inhibit the growth, proliferation, and metastasis of tumor cells in various ways and promote the apoptosis of tumor cells (You et al., 2018; Zhao et al., 2019b; Kim et al., 2019). Kim et al. (Kim et al., 2019) confirmed that Scutellaria Radix could promote apoptosis in non-small-cell lung cancer cells via induction of AMPK-Dependent Autophagy pathway. Zhao et al. (Zhao et al., 2019b) found that Scutellaria flavonoids effectively inhibited the malignant tumors of non-small-cell lung cancer in an id1-dependent manner. The study (Han et al., 2017; You et al., 2018) found that baicalin inhibited the survival, migration, and invasion of human non-small-cell lung cancer A549 and H1299 cells in a dose-dependent manner *in vitro*. Baicalin can inhibit the migration and invasion of non-small-cell lung cancer by activating motor (mammalian target of rapamycin) and SIRT1/AMPK signaling pathway. Hussain I et al. (Hussain et al., 2018) found that *Scutellaria baicalensis* could target the hypoxia-inducible factor-1α to inhibit the proliferation of ovarian cancer cells and enhance cisplatin efficacy in ovarian cancer patients. Baicalin can inhibit the invasion and metastasis of breast cancer by inhibiting the TGF-β1 signaling pathway, upregulating E-cadherin, and downregulating the expression of *p*-Smad3 and vimentin *in vivo* and *in vitro*. Baicalin is a safe and effective potential antitumor invasion and metastasis drug, which has great potential in improving the prognosis quality of tumor patients (Liu D. K. et al., 2020).

As for the research of *Scutellaria baicalensis* Georgi in treating gastric cancer, researchers have found that *Scutellaria baicalensis* Georgi can inhibit the proliferation and migration of human gastric cancer MGC-803 and SGC-7901 cells and induce their apoptosis (Avila-Carrasco et al., 2019; Miao et al., 2019; Zhang, 2019). Li et al. (Li et al. 2018) screened out the action target of *Scutellaria baicalensis* Georgi in inducing apoptosis of gastric cancer SGC-7901 cells through antibody chip. Researchers (Bai et al., 2017; Xu, 2017; Sun et al., 2018) found that *Scutellaria baicalensis* Georgi can regulate the expression of TLR8, HIF-1α, PDGFβ, and PTEN in gastric cancer cells, which can inhibit invasion, migration, and epithelial-mesenchymal transition of gastric cancer cells through NF-κB/Snail signaling pathway. Xiong et al. (Xiong et al., 2016) found that *Scutellaria baicalensis* Georgi can reverse 5-Fu resistance induced by hypoxia in gastric cancer patients. However, generally speaking, the antitumor experiments of *Scutellaria baicalensis* Georgi are mainly *in vitro*, and there is still much room to explore the potential mechanism of *Scutellaria baicalensis* Georgi against gastric malignant tumor. Although previous biological experiments have confirmed the inhibitory effect of *Scutellaria baicalensis* Georgi on gastric cancer cells, its active components, targeted regulation genes, and molecular mechanism of inhibiting gastric cancer are still unclear.

Network pharmacology is based on the similarity between drugs in structure and efficacy, and considering the interaction between target molecules and biological effector molecules in the body and through the joint analysis of disease-related genes, the regulation network of drug-component-target-disease is constructed. Traditional Chinese medicine compound refers to the mixture of two or more traditional Chinese medicines, and its regulatory network is more complicated. Network pharmacology emphasizes multichannel regulation of signal pathways, improves the therapeutic effect of drugs, and reduces toxic and side effects, thus increasing the success rate of clinical trials of new drugs and saving the cost of drug research and development.

In order to clarify the theoretical basis and potential molecular mechanism of *Scutellaria baicalensis* Georgi in the treatment of gastric cancer, this study analyzed the main effective chemical constituents of *Scutellaria baicalensis* Georgi by means of network pharmacology, explored its targeted regulation genes, and screened out the effective target gene sites for the treatment of gastric cancer. After constructing the interaction structure diagram of *Scutellaria baicalensis* Georgi acting on target genes to regulate the occurrence and development of gastric cancer, we further analyzed the possible signal pathways of these genes and further studied the molecular docking and visualization of the effective chemical components of *Scutellaria baicalensis* Georgi.

## Materials and Methods

### Screening of Effective Active Ingredients and Action Targets of *Scutellaria baicalensis* Georgi

Screening of effective active ingredients and action targets of *Scutellaria baicalensis* Georgi passed through the pharmacology database of Chinese medicine system and the analytical platform (TCMSP, Traditional Chinese medicine systems pharmacology database and analysis platform) was used to search the main chemical components and related target information of *Scutellaria baicalensis* Georgi in Chinese medicine system pharmacology database^1^. Taking oral bioavailability (OB) ≥ 30% and drug-likeness (DL) ≥ 0.18 as screening conditions, the drug targets were determined. Then, drug targets were screened by DrugBank (https://www.drugbank.ca/) database, and drug target genes were annotated by the uniprot^2^ database.

### Identification of *Scutellaria baicalensis* Georgi

Keywords “Gastric cancer; Stomach cancer; gastric carcinoma” were retrieved from GeneCards^3^, OMIM^4^, PharmGkb^5^ and TTD^6^ databases respectively, to obtain gastric cancer-related genes. The Online Wayne analysis tool^7^ was used to select the intersection of genes selected from five databases as the common target for the treatment of gastric cancer. The target gene of *Scutellaria baicalensis* Georgi against gastric cancer was obtained by the intersection of the drug target gene and gastric cancer target gene with the VennDiagram package of the R language.

### Constructions of Regulatory Network and Protein Interaction Network

The regulatory network of “*Scutellaria baicalensis* Georgi-active ingredient compound-gene target-gastric cancer” was mapped by software Cytoscape version 3.4.0. The Network Analyzer tool of Cytoscape software was used to calculate the topological properties of the network, then the basic attributes of the network were exported in CS V format and sorted according to the degree and median centrality of nodes, and the top 10 traditional Chinese medicine compounds and drug targets are selected. The target protein interaction network was constructed by using STRING database^8^, and the species was defined as “*Homo sapiens*.” Use Cytoscape plug-in CytoNCA to find the core genes in the PPI network.

### Gene Ontology (GO) and Kyoto Encyclopedia of Genes and Genomes (KEGG) Enrichment Analysis

The functional annotation of GO and enrichment analysis of KEGG were performed by using clusterProfiler package in R language.

### Molecular Docking

The SMILES number of the compound was got from PubChem^9^database, and the SMII of the compound was put into the SwissTargetPrediction^10^database to get the predicted target of each chemical component and download the sdf format compound. Chem3D software was used to convert the mol2 format, and the 2D structure was converted into a 3D structure. The crystal structures of proteins corresponding to the core genes were downloaded from pdb database^11^, the water molecules were deleted by PyMOL software, and hydrogen was added for pretreatment. Binding free energy is the interaction between small molecule ligand and protein receptor. In this study, the binding free energy (G) is calculated by the MM-GBSA method, and the result is expressed as a negative value. The smaller the G is, the closer the ligand binds to a protein. AutoDock Vina was used for molecular docking, and Discovery Studio Visualizer was used for visualization of results.

### Effect of *Scutellaria baicalensis* Georgi on Proliferation and Migration of Gastric Cancer Cells

Human gastric cancer cell MKN-45 was inoculated into a six-well plate. The baicalin group in the experimental group was divided into four groups according to its concentration: 160 μmol/L, 80 μmol/L, and 40 μmol/L. A negative control group was established (only 0.9% NaCl was added). After treatment for 24 h, the cell morphology of each group was observed under an inverted optical microscope. The 24-well plate was inoculated according to 10,000 cells/well, and three multiple-well and negative control groups were set up. After adding drugs for 24 h, an MTT reagent (5 μg/ml) was added, and the culture was continued for 4 h. After shaking for 2 s, the 490 nm reading value was read on the microplate reader, and the cell proliferation rate was calculated with reference to the control group.

We used a ruler to assist the fixed comparison and marked a line for every 0.5–1 cm behind the six-hole plate with a marker pen, with at least five lines passing through each hole. About 5 × 10^5^ cells were added to each well, and a complete medium was added so that the cells could cover the wells overnight. The next day, when the cells were fully under the microscope, we used the gun head close to the edge of the ruler and scratched along the horizontal line behind the six-well plate. During this period, pay attention to keeping the gun head perpendicular to the plate surface and not inclined. The cells were washed with sterile PBS three times to remove exfoliated cells and impurities and then cultured in a serum-free medium. The six wells were plated in a cell incubator for culture. At 0 and 24 h, the cells were taken out and photographed under a microscope. The Transwell method was used to detect the migration ability of the cells in the four groups.

50 μL 10 g/L BSA of serum-free culture medium was added to each hole, 37°C, 30 min. Trypsin was used to digest cells, which were then flushed with PBS 1–2 times to remove the effect of serum. Cells were resuspended with serum-free medium, with a cell density of 5*10^5^/ ml. Cell suspension 200 μL was taken and added into the Transwell chamber. Under the 24-hole culture plate, 600 μL containing 10% FBS medium was added indoors. The plate was placed in a CO_2_ incubator at 37°C and cultured for 24 h. Chamber was removed and washed twice with PBS, and the cells on the upper layer of the microporous membrane were carefully wiped with a cotton swab. Cells were fixed for 20 min with 4% paraformaldehyde in the 24-well plate and stained with crystal violet solution for 15 min. Photograph was taken under an inverted microscope.

### Western Blotting

Gastric cancer cells were divided into *Scutellaria baicalensis* group, oxaliplatin group, and control group. *Scutellaria baicalensis* group was treated with 40 μg/ml *Scutellaria baicalensis*, oxaliplatin group was treated with the same concentration of oxaliplatin, and the control group was treated with normal saline. All three groups were treated for 24 hours. Western blotting was used to detect the expression of ESR1, FOS and Tubulin in the three groups.

## Results

### Construction of the “Immune Target-Traditional Chinese Medicine-Compounds-Disease” Network

**FIGURE 1:**
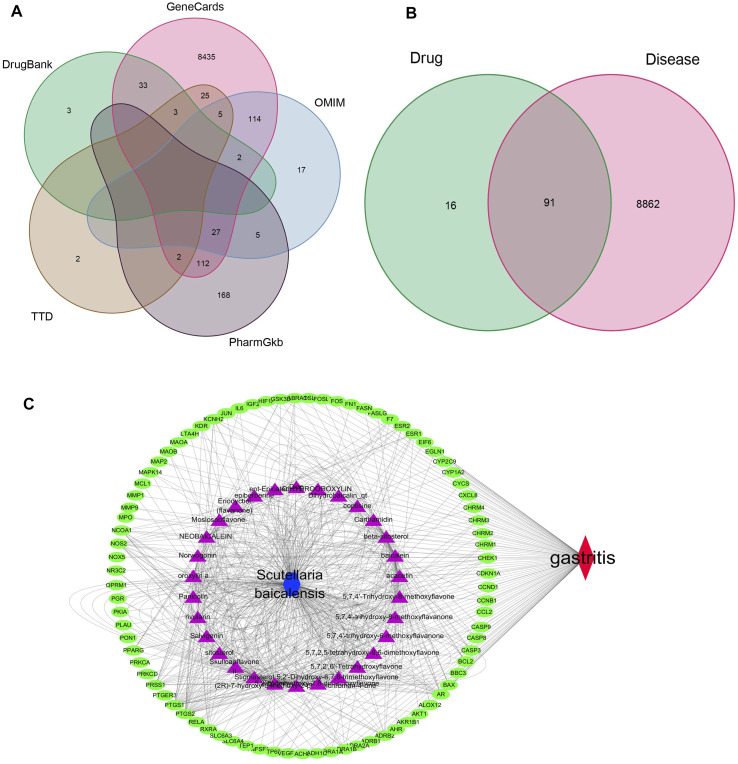
**(A)** The Venn diagram of the intersection of gastric cancer-related genes obtained from five databases. **(B)** Venn diagram of the intersection of target genes of *Scutellaria baicalensis* Georgi and gastric cancer-related genes. **(C)** The network map of “*Scutellaria baicalensis* Georgi-compound-drug action target-disease.”

The TCMSP was used to screen the recommended active ingredients of TCM compounds, with OB ≥ 30% and DLOB≥0.18 as the screening conditions. 30 active drugs and 107 corresponding action targets were annotated by the DrugBank database. 8,953 genes associated with gastric cancer were screened from five databases. A total of 328 genes were obtained as common targets for the treatment of gastric cancer (Figure 1A). After that, 91 drug targets of *Scutellaria baicalensis* were obtained by intersecting with gastric cancer target genes (Figure 1B). The main active components and corresponding gene targets of *Scutellaria baicalensis* were shown in Supplementary Table S1. The nodes with large values represent the key role in the network, which may be the key compounds or targets. The compounds with high recommendation value in the network mainly have the highest acacetin, wogonin, baicalein, Salvigenin, and Moslosooflavone value ranking of gastric cancer disease-related targets mainly NOS2, PTGS1, PTGS2, DPP4, and so on. The interaction between active compounds in *Scutellaria baicalensis* and related targets of gastric cancer is shown in Supplementary Table S2. Cytoscape software was used to construct the network map of “traditional Chinese medicine-compound-drug action target-disease” (gastric cancer-91 antigastric cancer target-30 compounds-*Scutellaria baicalensis*) (Figure 1C).

### PPI Network Constructions and Function Enrichment Analysis

**FIGURE 2:**
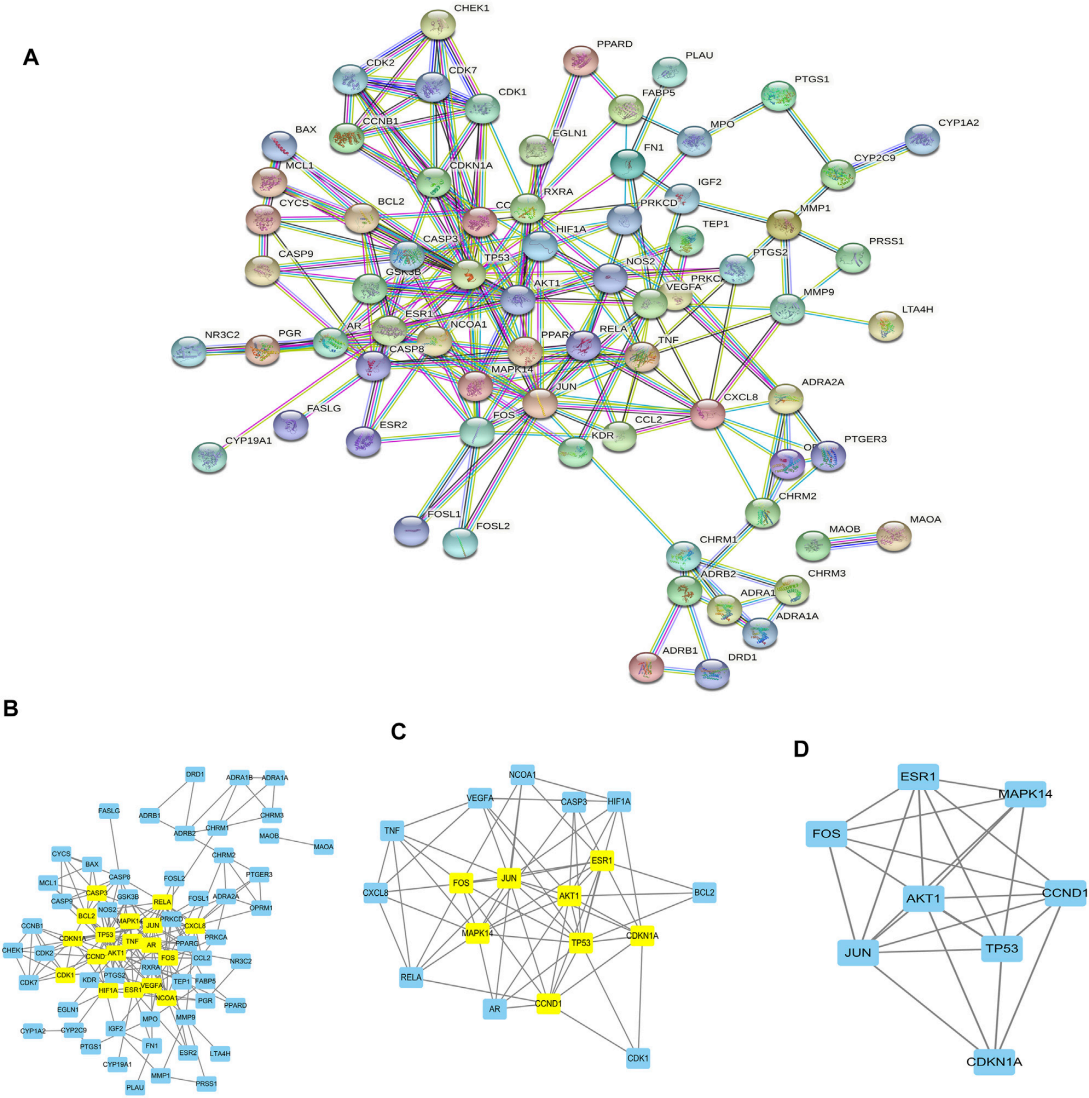
**(A)** Diagram of protein interaction network of gastric cancer target genes acted by *Scutellaria*. **(B)** Topological network of gastric cancer target genes acted by *Scutellaria baicalensis* Georgi. **(C)** Further screening of target genes for drug action. **(D)** Eight core drug target genes of *Scutellaria baicalensis* Georgi.

**FIGURE 3:**
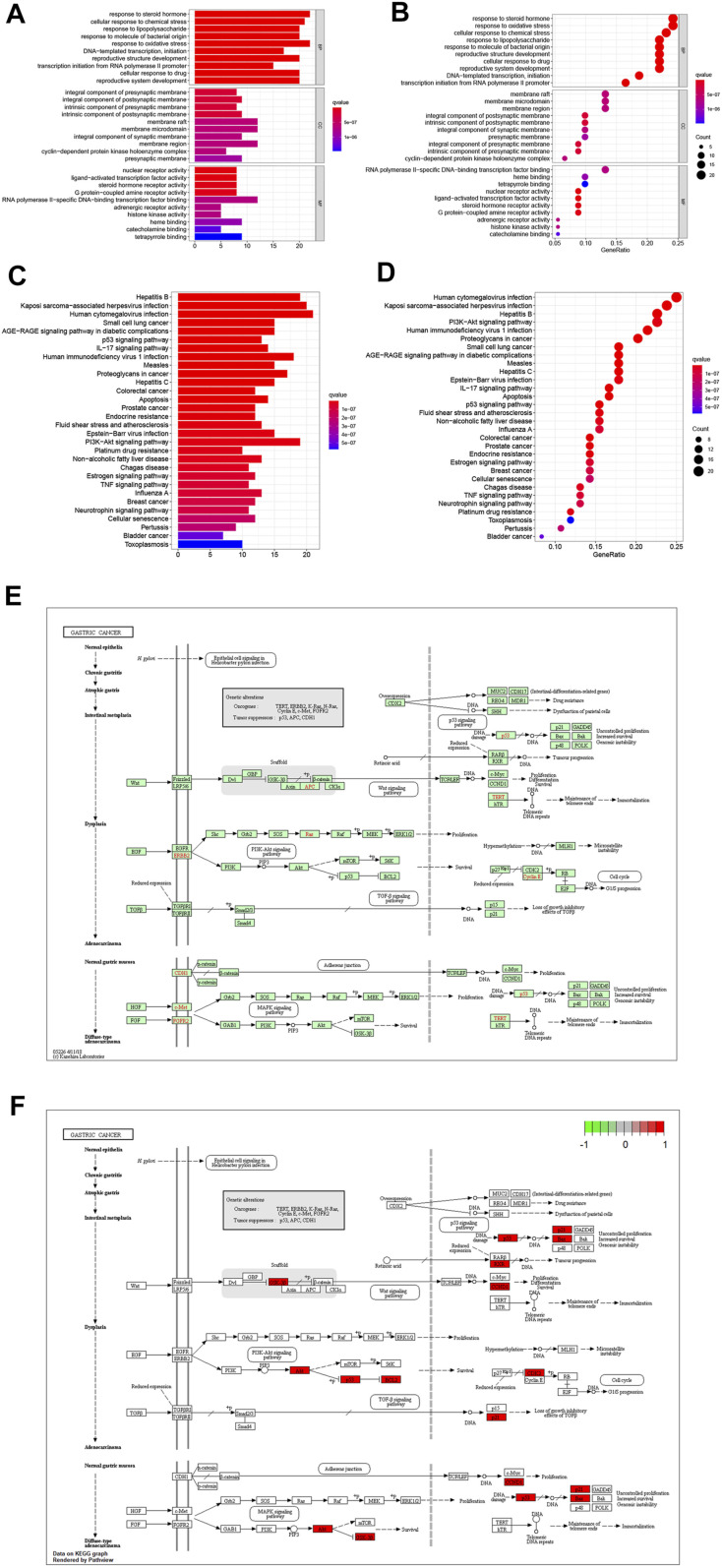
**(A)** Bar graph of GO enrichment analysis of target genes of *Scutellaria baicalensis* in antigastric cancer. **(B)** Bubble graph of GO enrichment analysis of target genes of *Scutellaria baicalensis* in antigastric cancer. **(C)** KEGG enrichment of target genes of *Scutellaria baicalensis* in antigastric cancer Bar graph of analysis. **(D)** Bubble chart of KEGG enrichment analysis of target genes of *Scutellaria baicalensis* Georgi on antigastric cancer; 3E: KEGG signaling pathway of *Scutellaria baicalensis’* antigastric cancer effect. **(F)** Key molecules of *Scutellaria baicalensis* antigastric cancer effect.

The target genes of gastric cancer acted by *Scutellaria baicalensis* Georgi were analyzed by protein interaction (Figure 2A), then the core parts of the network were analyzed by CytoNCA, and three core topological networks were obtained. Eight core genes were screened out: MAPK14, JUN, FOS, AKT1, CDKN1A, TP53, ESR1, and CCND1 (Figures 2B–D). GO analysis found that the main biological functions of *Scutellaria baicalensis* Georgi in treating gastric cancer include responses to steroid hormones, responses to oxidative stress, development of the reproductive system, cellular responses to drugs, development of reproductive structures, responses to bacteria-derived molecules, responses to lipopolysaccharides, and cellular responses to chemical stress (Figures 3A,B). The treatment of gastric cancer by *Scutellaria baicalensis* Georgi mainly involves human cancer infection, cancer in cancer, human immunodeficiency virus 1 infection, PI3K/Akt signaling pathway, hepatitis B, Kaposi sarcoma-associated herpesvirus infection, and other signal pathways (Figures 3C–F).

### Molecular Docking of the Core Effective Components of *Scutellaria baicalensis* Georgi in the Treatment of Gastric Cancer

**FIGURE 4:**
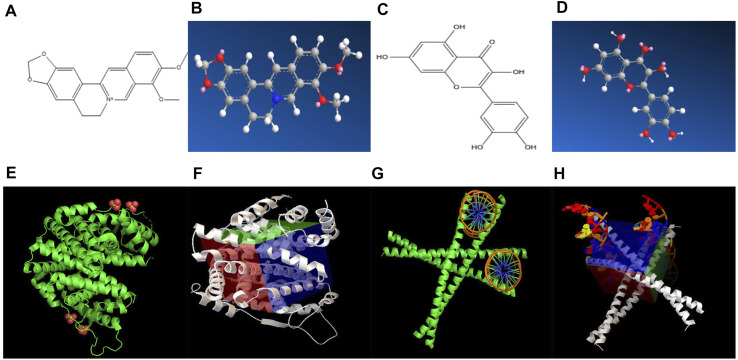
**(A)** 2D chemical structure formula of ESR1 target molecule. **(B)** 3D chemical structure formula of ESR1 target molecule. **(C)** 2D chemical structure formula of FOS target molecule. **(D)** 3D chemical structure formula of FOS target molecule. **(E)** ESR1 target molecule and compound molecular docking diagram of *Scutellaria baicalensis*. **(F)** Active pocket for docking ESR1 target and compound molecules of *Scutellaria baicalensis*. **(G)** Docking diagram of FOS target and compound molecules of *Scutellaria baicalensis*. **(H)** Active pocket for docking FOS target and compound molecules of *Scutellaria baicalensis*.

The effective target genes and 2D structure were obtained from the SwissTargetPrediction database, and the Chem3D software was used to convert the mol2 format to obtain the 3D structure of the target gene (Figures 4A–D). AutoDock Vina was used to dock the core active ingredients of *Scutellaria baicalensis* Georgi (the top 10 compounds in the network) with eight gene targets, and it was found that ESR1 and FOS had good binding free energy and docking score (Figures 4E–H). The docking fraction of FOS is −14.200 and the binding free energy is −27.893 kcal/mol. The docking fraction of ESR1 is −15.833 and the binding free energy is −30.001 kcal/mol. The docking score of FOS and ESR1 is better than that of the original ligand, suggesting that *Scutellaria baicalensis* Georgi has a prominent effect on these two targets. The binding energy of FOS and ESR1 is close to 29.3 kJ mol^-1^, which indicates that the two targets are closely bound to the active compounds of *Scutellaria baicalensis* Georgi, and the receptor and ligand have a good affinity.

### Experimental Studies on the Effect of *Scutellaria baicalensis* Georgi on the Proliferation and Migration of Gastric Cancer Cells

**FIGURE 5:**
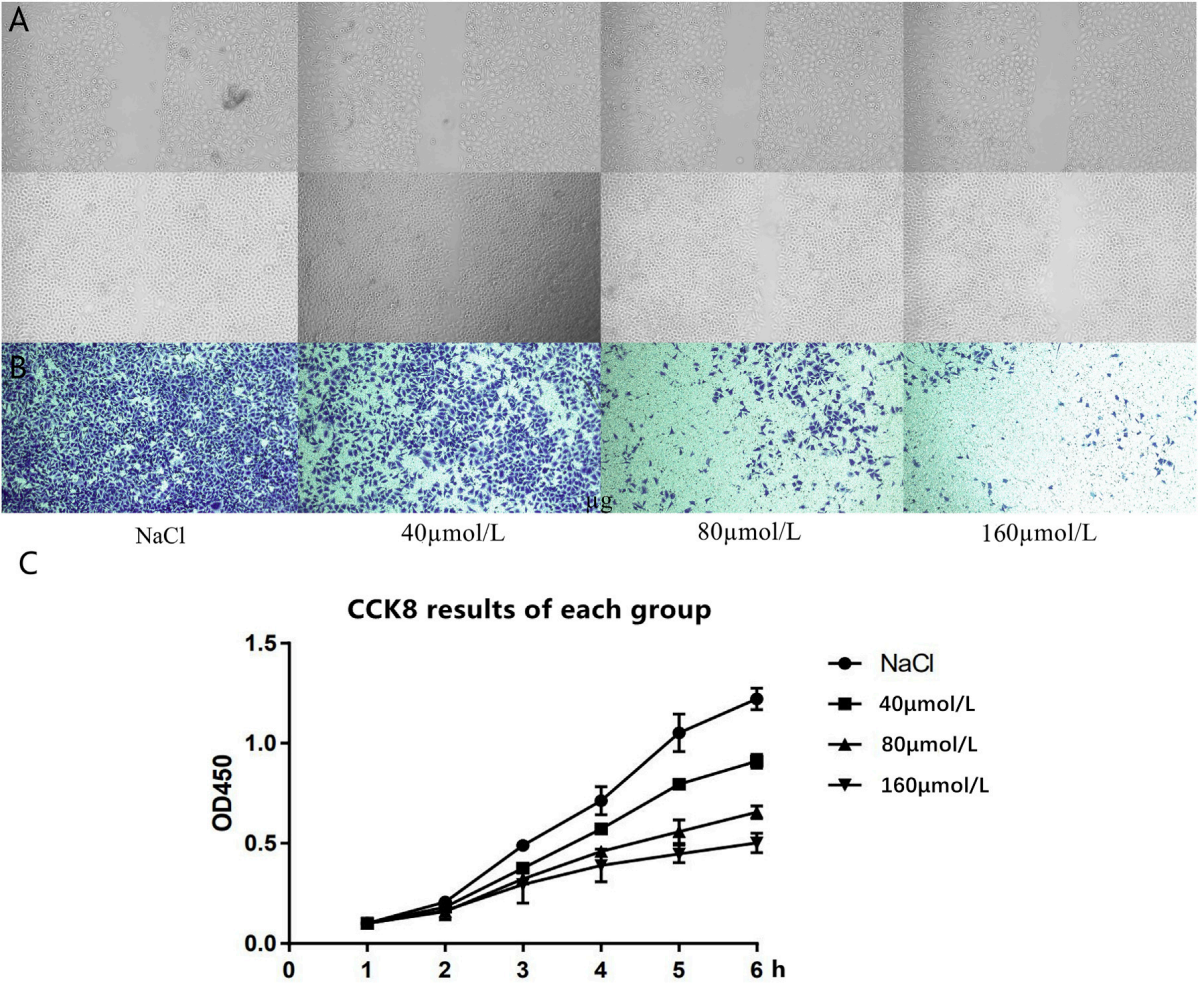
**(A)** The cell scratch test results of the four groups of cells at 0 and 24 h. **(B)** Transwell experiment detects the cell migration results of the four groups of cells at 24 h. **(C)** CCK8 experiment results of each group.

The results of the scratch experiment showed that there was no significant difference in the scribing width of the four groups of cells at 0 h, and the groups were comparable; at 24 h, compared to the control group, the scribing widths of the 80 and 160 μmol/L groups were significantly shorter (Figure 5A). Transwell experimental results revealed that, compared with the control group, the number of cells in the 40 μmol/L group was reduced, and the difference was statistically significant (*p* < 0.05). Compared with the 40 μmol/L group, the number of cells in the 80 and 160 μmol/L groups was significantly reduced, and the number of cells in the 160 μmol/L group was less than that in the 80 μmol/L group (*p* < 0.05) (Figure 5B). The CCK8 experiment results of the four different drug concentration treatment groups showed that, compared with the NaCl control group, the proliferation ability of the cells in the 40 μmol/L group was significantly reduced, the cell proliferation ability continued to decrease gradually in the 80 μmol/L group and the 160 μmol/L group (Figure 5C), and the difference was statistically significant.

### Western Blotting of the Expression of Target Genes in Tumor Cells

**FIGURE 6:**
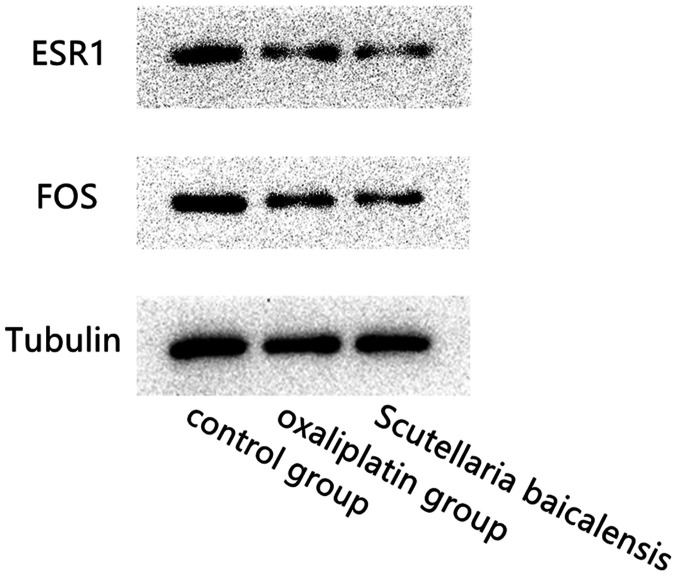
Western blotting results show the comparison of the expression levels of ESR1, FOS and Tubulin genes in *Scutellaria baicalensis* group, oxaliplatin treated group and the control group.

Western blotting results showed that compared with the control group, ESR and FOS expression levels in the *Scutellaria baicalensis* group and Oxaliplatin group were significantly lower. There was no significant difference in the expression of ESR1 and FOS genes between the *Scutellaria baicalensis* group and the Oxaliplatin group. And the reference gene Tubulin was stably expressed among the three treated groups (Figure 6).

## Discussion

In this study, potential drug targets of *Scutellaria baicalensis* Georgi were screened through TCMSP, PharmGkb, TTD, and DrugBank databases, these targets were mapped with gastric cancer targets obtained in the DisGeNET database, and 91 genetic targets of *Scutellaria baicalensis* Georgi against gastric cancer were obtained. The protein–protein interaction network was analyzed and mapped with the help of the STRING database and Cytoscape 3.7.1. Core genes in PPI topological network were found by CytoNCA, and eight core targets including MAPK14, JUN, FOS, Akt1, CDKN1A, TP53, ESR1, and CCND1 were obtained. These eight target genes interact most closely with other targets, suggesting that they may be the core target genes for *Scutellaria baicalensis* Georgi to exert pharmacological effects. AutoDock Vina was used for molecular docking between the core active ingredients and gene targets of *Scutellaria baicalensis* Georgi. Among the eight core targets, ESR1 and FOS are the key gene targets with the most effects on the chemical ingredients of *Scutellaria baicalensis* Georgi. It is generally believed that the binding energy value is less than 29.3 kJ mol^-1^, which indicates that receptor and ligand have a good affinity, and ESR1 and FOS have good binding free energy and docking fraction (docking fraction of FOS is −14.200, and binding free energy is −27.893 kcal/mol); ESR1 has a docking score of −15.833 and binding free energy of −30.001 kcal/mol, which may be a key drug target for treating gastric cancer.

ESR1 mainly encodes estrogen receptor á, which is closely related to breast cancer and endometrial cancer, and some studies have shown that the estrogen receptor is also related to gastric cancer (Ge et al., 2018). Estrogen receptor á can be divided into three types, namely, ERá66, ERá46, and ERá36. A joint experiment on tamoxifen and naringenin shows that drugs can inhibit the proliferation of breast cancer cells by downregulating the expression of ERá66 or upregulating the expression of ERá36 (Xu et al., 2018). Activator protein-1 (AP-1) is composed of c-JUN protein and c-FOS protein family members. It is an important transcription factor belonging to the bZIP class of DNA-binding proteins in cells. AP-1 can regulate many inflammatory factors such as IL-1, IL-6, IL-8, MCP-1, ICAM-1, and VCAM-1, initiate gene transcription, and play an important role in cell proliferation, differentiation, and apoptosis. The activity of AP-1 is regulated by a variety of nuclear factors, and the monomers also have mutual promotion or antagonism. AP-1 can make physiological or pathological responses to various stimuli such as stress, radiation, or growth signals and participates in the development of cells. Processes such as proliferation, differentiation, and transformation play an important role in tumor formation, metastasis, and invasion. *Scutellaria baicalensis* may affect the expression of AP-1 protein by changing the expression of the FOS gene through chemical stimulation of cells, thereby changing the proliferation and apoptosis of tumor cells by regulating the AP-1 signaling pathway. FOS gene encodes leucine zipper protein, which can dimerize with JUN family proteins to form transcription factor complex AP-1. Therefore, FOS protein is considered as the regulator of cell proliferation, differentiation, and transformation. Liu et al. (Liu K. et al., 2020) found that the polymorphism of the FOS-like antigen 1 gene is related to the susceptibility to gastric cancer. Zhang et al. (Zhang et al., 2011) found that the loss of c-FOS expression is associated with cancer progression, lymph node metastasis, lymphatic infiltration, and short survival, suggesting that the loss of c-FOS expression in gastric cancer cells may be related to cancer and poor prognosis. Zhu et al. (Zhu et al., 2010) found that miR-222 can regulate FOS gene expression in a targeted way, thus affecting the proliferation and migration of tumor cells.

Our research found that the active compounds such as quercetin, kaempferol, beta-sitosterol, and stigmasterol (R)-canadine in *Scutellaria baicalensis* Georgi play an antigastric cancer role by binding ESR1, FOS, and other genes. In order to prove that *Scutellaria baicalensis* Georgi can control the progression of gastric cancer by inhibiting the proliferation and migration of tumor cells, we added *in vitro* experiments to verify it. CCK8 experiment proved that *Scutellaria baicalensis* Georgi could inhibit the proliferation of gastric cancer cells *in vitro*, and the degree of inhibition was positively correlated with drug concentration. The Transwell experiment confirmed that *Scutellaria baicalensis* Georgi can inhibit the migration of gastric cancer cells *in vitro*, and the degree of inhibition is positively correlated with drug concentration. However, our study also has certain limitations: it did not add standard gastric cancer treatment drugs for comparison to determine the antigastric cancer efficacy of *Scutellaria baicalensis* Georgi, did not verify the difference in gene expression of the two main target genes before and after treatment with *Scutellaria baicalensis* Georgi, and did not further elaborate on the correlation mechanism.

## 4 Conclusion

In this study, the active compound components, drug action targets, and gastric cancer-related gene targets of *Scutellaria baicalensis* Georgi were explored by using network pharmacological analysis technology, the interaction network of *Scutellaria baicalensis* Georgi antigastric cancer gene targets was constructed, and the key components and eight core action targets of *Scutellaria baicalensis* Georgi antigastric cancer were screened out. The binding activity between target and drug was simulated by molecular docking, which further confirmed that ESR1 and FOS genes were the key drug targets. Furthermore, the effect of *Scutellaria baicalensis* Georgi on gastric cancer was verified by *in vitro* cell experiments.

## Data Availability

The original contributions presented in the study are included in the article/Supplementary Material; further inquiries can be directed to the corresponding author.

## Footnotes

1
http://tcmspw.com/tcmsp.php

2
https://www.uniprot.org/

3
https://www.genecards.org

4
http://omim.org/

5
https://www.pharmgkb.org/

6
http://db.idrblab.net/ttd/

7
http://bioinformatics.psbe/webtools/venn/

8
https://String-db.org/

9
https://pubchem.ncbi.nlm.nih.gov

10
http://swisstargetprediction.ch/

11
http://www.rcsb.org/
